# Editor’s Outlook

**Published:** 1893-05

**Authors:** 


### EDITOR'S OUTLOOK.

### A WORD ABOUT OURSELVES.

In presenting the May number of the JOURNAL to the public, the editor feels it his duty in this formal manner, to say a few words to its large army of readers. It is, however, at any time, difficult to be personal without incurring the charge of being egotistical, and, although the present occasion may offer an apology for giving utterance to our feelings on reviewing the labors connected with the JOURNAL for the last forty years, we defer, for once at least, to the already expressed testimony of others. As journalists, seeking to promote the higher interests of men, striving to be watchful for them, and faithful in all we say, and to furnish them periodically with subjects, coming home to their bosoms and their business, and to their firesides, we are well aware that *their* verdict must determine how far we have met our engagements.

And it would be alike ungrateful to the public, as unjust to ourselves, were we to overlook, or in any degree depreciate the encouragement which has been extended to us, or the very favorable opinions, all unsolicited, which they have so plenteously expressed.

A retrospect glance at the JOURNAL's course we find that we have had a word for almost every one. The minister in his pulpit and the worshipper in his pew, the merchant in his warehouse, and the workman at his toil, the father executing his parental trust and the mothor mindful of her influence and responsibility, those who have their partialities for poetry and those who have their preferences for biography and, finally, the student of history and the student of the Bible.

Never in its history has the JOURNAL been in a more prosperous condition than it is to-day ; it has outgrown its present quarters, and in consequence has found a new home ; it is alive and on the alert for all new developments in the medical world especially, and its regular readers are found in every part of the globe.

The principles which have characterized the JOURNAL for forty years and won for it the confidence and esteem of its readers will be faithfully and vigorously upheld. The publishers have many new plans to be utilized and carried out, and will endeavor, sparing no pains and expense, to make the JOURNAL an ideal family magazine. Our staff of contributors, we need scarcely say, is and will be, composed of men for the times and having many pens of admitted ability ready when occasion calls, the readers of the JOURNAL may confidently anticipate that its pages shall be occupied with matters bearing on the more momentous interests of the times Feeling grateful for the kind hints and suggestions of numerous friends, we propose, as we have said heretofore to make many improvements in the JOURNAL which will, we trust, be appreciated, and while due attention will be paid to matters concerning the health of the home, we shall also endeavor to work out more fully our design to render it a suitable periodical for the family fireside.

---

Where are you going this summer for health and rest ? is the question that is agitating the people's minds just now, and in consequence, the publishers of the JOURNAL have decided to open a Recreation Bureau in connection with the magazine, so that if any of its readers want enlightenment regarding any summer resort in the United States or Canada, how to reach it, information about any of the hotels, their rates, location, etc., simply address the Recreation Department, HALL'S JOURNAL OF HEALTH, Express Building, 23 Park Row, New York.

All information furnished free.

---

Why we make such a splendid and valuable offer of Chambers Encyclopedia in connection with the JOURNAL, can be readily seen. We want to swell our subscription list to *Thirty thousand* copies by September 1. It can be accomplished if every reader and subscriber of the JOURNAL will aid us.

This offer is especially made to the JOURNAL, and in order to ensure promptness in receiving the Encyclopedia, send in your subscriptions early.

---

Please bear in mind that the JOURNAL has moved into new commodious offices in the Express Building, 23 Park Row, where the publishers will be pleased to meet the magazine's many friends.

---

If you do not find the JOURNAL on any of the news stands, please inform us either by person or mail, and we will gladly furnish you a copy.

